# PD-L1 expression and association with genetic background in pheochromocytoma and paraganglioma

**DOI:** 10.3389/fonc.2022.1045517

**Published:** 2022-11-11

**Authors:** Katerina Hadrava Vanova, Ondrej Uher, Leah Meuter, Suman Ghosal, Sara Talvacchio, Mayank Patel, Jiri Neuzil, Karel Pacak

**Affiliations:** ^1^ Section on Medical Neuroendocrinology, Eunice Kennedy Shriver National Institute of Child Health and Human Development, National Institutes of Health, Bethesda, MD, United States; ^2^ School of Pharmacy and Medical Science, Griffith University, Southport, QLD, Australia; ^3^ Institute of Biotechnology, Czech Academy of Sciences, Prague-West, Czechia; ^4^ Faculty of Science and 1st Medical Faculty, Charles University, Prague, Czechia

**Keywords:** neuroendocrine tumors, immunotherapy, pheochromocytoma, paraganglioma, programmed death ligand 1

## Abstract

Metastatic pheochromocytomas and paragangliomas (PPGLs) are rare neuroendocrine tumors associated with poor prognosis and limited therapeutic options. Recent advances in oncology-related immunotherapy, specifically in targeting of programmed cell death-1 (PD-1)/programmed death-ligand 1 (PD-L1) pathways, have identified a new treatment potential in a variety of tumors, including advanced and rare tumors. Only a fraction of patients being treated by immune checkpoint inhibitors have shown to benefit from it, displaying a need for strategies which identify patients who may most likely show a favorable response. Building on recent, promising outcomes in a clinical study of metastatic PPGL using pembrolizumab, a humanized IgG4κ monoclonal antibody targeting the PD-1/PD-L1 pathway, we examined PD-L1 and PD-L2 expression in relation to oncogenic drivers in our PPGL patient cohort to explore whether expression can predict metastatic potential and/or be considered a predictive marker for targeted therapy. We evaluated RNA expression in the NIH cohort of 48 patients with known genetic predisposition (sporadic; pseudohypoxia: *SDHB*, *VHL*, *EPAS1*, *EGLN1;* kinase signaling: *RET*, *NF1*) and 6 normal medulla samples (NAM). For comparison, 72 PPGL samples from The Cancer Genome Atlas (TCGA) were used for analysis of gene expression based on the variant status (pseudohypoxia: *SDHB*, *VHL*, *EPAS1*, *EGLN1*; kinase signaling: *NF1*, *RET*). Expression of PD-L1 was elevated in the PPGL cohort compared to normal adrenal medulla, aligning with the TCGA analysis, whereas PD-L2 was not elevated. However, expression of PD-L1 was lower in the pseudohypoxia cluster compared to the sporadic and the kinase signaling subtype cluster, suggesting that sporadic and kinase signaling cluster PPGLs could benefit from PD-1/PD-L1 therapy more than the pseudohypoxia cluster. Within the pseudohypoxia cluster, expression of PD-L1 was significantly lower in both *SDHB*- and non-*SDHB*-mutated tumors compared to sporadic tumors. PD-L1 and PD-L2 expression was not affected by the metastatic status. We conclude that PD-L1 and PD-L2 expression in our cohort of PPGL tumors was not linked to metastatic behavior, however, the presence of PPGL driver mutation could be a predictive marker for PD-L1-targeted therapy and an important feature for further clinical studies in patients with PPGL.

## Introduction

Immune checkpoint inhibitors have been shown to benefit patients affected by diverse cancers, e.g. melanoma (1, 2), lung cancer (3, 4), and renal cancer (5). However, there are also downsides to these therapies, such as high cost, toxicity, and relatively low response rate. Strategies to adequately identify patients who are likely to show favorable responses to immune checkpoint inhibitors are needed. Recently, promising outcomes related to immunotherapy have been documented in patients with advanced rare cancers, including a cohort of 9 patients with pheochromocytoma and paraganglioma (PPGL) (6). Metastatic PPGLs are rare neuroendocrine tumors often associated with poor prognosis and limited therapeutic options (7–9). In a clinical study with pembrolizumab, a humanized IgG4κ monoclonal antibody targeting the PD-1/PD-L1 pathway, the authors concluded that the clinical benefit rate was 75% (95% CI, 35% to 97%) and non-progression rate at 27 weeks was 43% (three of seven evaluated patients; 95% CI, 10% to 82%) (6). Additionally, the treatment with nivolumab (anti-PD-1 antibody) and ipilimumab (anti-CTLA-4 antibody) showed benefit in a case of rare metastatic pheochromocytoma, supporting the ongoing use of immunotherapy in rare tumors in clinical trials (10). While current data provide a rationale for the use of pembrolizumab or other immune checkpoint inhibitors in these tumors, the possibility of patient response based on molecular characterization of the tumor was not explored. Since susceptibility gene mutations are prevalent in patients with PPGL, molecular characterization and correlation necessitate consideration for precision medicine in these malignancies (9, 11). Establishment in the relation of *PD-L1*/*PD-L2* expression to driver mutations in PPGL patients may save the patients from improper treatment as well as unnecessary biopsy that may trigger adverse reactions due to catecholamine release.

PD-L1 expression is one of the predictors to determine the potential response by blockade of immune checkpoints as cancer therapy (12–14). In the aforementioned clinical study, patients with higher PD-L1 score (H-score >42.5) were more likely to survive and be progression-free for 27 weeks after pembrolizumab treatment compared to those with lower H-score (6). Studies on PD-L1 status in PPGL patients remain limited where the largest study on the expression of PD-L1 and PD-L2 found it to be present in only 18% and 16% in a cohort of 100 patients with PPGL, respectively, but only PD-L2 levels in this cohort correlated with metastatic behavior and shorter survival (15). Contrary to those findings, a study by Guo et al. indicated PD-L1 to be a metastatic marker for PPGL patients (16). Notwithstanding these findings, more studies are needed to extend our knowledge on the PD-L1/PD-L2 expression in separate patient cohorts to better predict the outcome immune checkpoint inhibitors. Given these discrepancies and the emerging concept of oncogenic drivers as promoters of immunosuppressive environment, here we explored if the *PD-L1* and *PD-L2* expression in our PPGL cohort can predict malignancy and/or be a predictive marker for PD-1/PD-L1 targeted therapy in PPGL.

## Materials and methods

### Patients

The study protocol was approved by the National Institutes of Health Institutional Review Board (NIH Protocol 00-CH-0093), and all patients provided written informed consent under this protocol. Normal adrenal medulla (NAM, n=6) and tumor tissues from our PPGL cohort (n=48) were used for the analysis. PPGL cohort included 32 patients with pheochromocytoma (PCC), 14 patients with paraganglioma (PGL) and 2 patients presented with both PCC and PGL at the time of diagnosis (PCC+PGL). Clinical details of patients included in this cohort are shown in **Supplementary Table 1**. Based on the genetic testing for known PPGL predisposing genes, twelve patients were considered as sporadic with no pathogenic variant identified. Within the pseudohypoxia cluster (n = 25), the most prevalent pathogenic germline variants were in *SDHB* (n = 14), followed by *VHL* (n = 8), and *EGLN1* (n = 1). This cluster included 2 additional patients with somatic *EPAS1* mutation. For the kinase signaling cluster (n = 11), pathogenic germline variants in *RET* (n = 7) and *NF1* (n = 4) were present. Metastatic disease was identified in 21 patients. All patients underwent surgery as the primary treatment of their tumors. One patient with metastatic disease received radiation therapy, chemotherapy, and immunotherapy (nivolumab and ipilimumab), and one patient received Iodine-131-meta-iodobenzylguanidine therapy (^131^I-MIBG) prior to tumor resection.

### The cancer genome atlas analysis

TCGA RNA-Seq V2 data for the expressions of PD-L1 (*CD274*) and PD-L2 (*PDCD1LG2*) genes in tumors with matched gene mutations (n = 72, pseudohypoxia: *SDHB*, *VHL*, *EGLN1*, *EPAS1*; kinase signaling: *NF1*, *RET*) to the study cohort and NAM samples (n = 3) were downloaded from the Genomic Data Commons (GDC) portal in the form of counts data processed using HTSeq-count pipeline. In the pseudohypoxia cluster, tumors with mutations in *SDHB* (n = 17), *VHL* (n = 10), *EPAS1* (n = 8), and *EGLN1* (n = 1) were included. The kinase signaling cluster comprised tumors with mutations in *NF1* (n = 22) and *RET* (n = 14). Expression of *PD-L1* and *PD-L2* was normalized to log counts per million (logCPM) using edgeR package.

### Gene expression

RNA from tumors and NAM was isolated with PureLink RNA minikit (Invitrogen, Walham, MA) and converted into cDNA High-Capacity cDNA Reverse Transcription Kit (Applied Biosystem, Walham, MA). Quantitative real-time PCR was run with PowerUp SYBR Green Master Mix (Applied Biosystems) and genes for PD-L1 (*CD274*, F: TGC CGA CTA CAA GCG AAT TAC TG, R: CTG CTT GTC CAG ATG ACT TCG G), PD-L2 (*PDCD1LG2*, F: CTC GTT CCA CAT ACC TCA AGT CC, R: CTG GAA CCT TTA GGA TGT GAG TG), and Ki-67 (*MKI67*, F: GAA AGA GTG GCA ACC TGC CTT C, R: GCA CCA AGT TTT ACT ACA TCT GCC) were evaluated to *ACTIN* (F: CAC CAT TGG CAA TGA GCG GTT C, R: AGG TCT TTG CGG ATG TCC ACG T). Data are expressed as ratio to calibrator.

### Statistical analysis

Data are expressed as the mean ± standard error of means. Data were evaluated by one-way ANOVA with multiple comparison *post-hoc* Tukey correction in GraphPad Prism (GraphPad Software, La Jolla, CA, USA) and Mann–Whitney non-parametric test. Data are considered statistically significant at p < 0.05.

## Results

**Figure 1:**
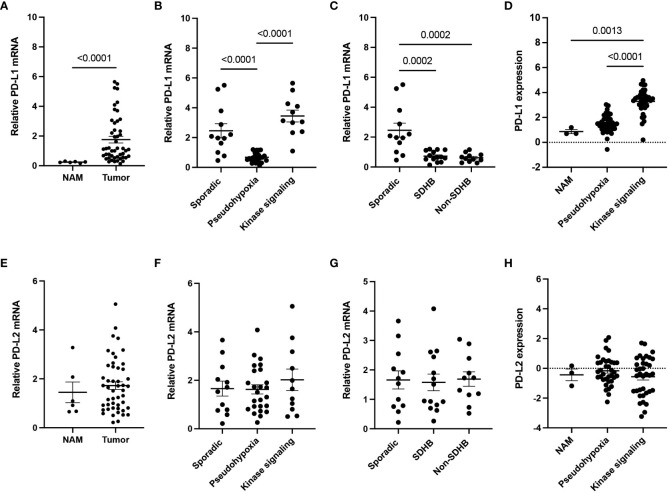
*PD-L1* and *PD-L2* expressions in PPGL cohort. **(A)** Expression of *PD-L1* (*CD274*) in PPGL tissue compared to normal adrenal medulla (NAM). **(B)** Expression of *PD-L1* in the pseudohypoxia and kinase signaling subtype cluster. **(C)** Expression of *PD-L1* within pseudohypoxia clusters based on *SDHB* mutation in our representative cohort (non-*SDHB* group comprises *VHL* and *EPAS1* mutations). **(D)** Expression of *PD-L1* in subtype clusters from TCGA. **(E)** Expression of *PD-L2* (*PDCD1LG2*) in PPGL tissue compared to normal adrenal medulla (NAM). **(F)** Expression of *PD-L2* in the pseudohypoxia and kinase signaling subtype cluster. **(G)** Expression of *PD-L2* within pseudohypoxia cluster based on *SDHB* mutation in our representative coho (non-*SDHB* group comprises *VHL* and *EPAS1* mutations). **(H)** Expression of *PD-L2* in subtype clusters from TCGA.

Gene expression of PD-L1 in our cohort of 48 patients with PPGLs was significantly elevated in the tumor tissue (n = 48) when compared to NAM samples (n = 6) (p<0.0001) (**Figure 1A**), suggesting tumor cell-immune cell interactions. To further elicit better predictors amongst the range of *PD-L1* expression in tumor tissues, we grouped the samples based on three genetic backgrounds: sporadic cases, pseudohypoxia cluster (*SDHB*, *VHL*, *EGLN1*, *EPAS1*) and kinase signaling cluster (*RET*, *NF1*). The pseudohypoxia cluster showed significantly decreased expression of *PD-L1* when compared to both sporadic and kinase signaling cluster (p < 0.0001) (**Figure 1B**). The pseudohypoxia-associated cluster was further divided into groups based on the presence of mutations either in the Krebs cycle-related genes or VHL/EPAS1-related genes. Both *SDHB*-mutated tumors and non-*SDHB*-mutated tumors (*VHL*- and *EPAS1*-mutated tumors), representing the pseudohypoxia cluster relative to the presence of mutations in the Krebs cycle enzyme succinate dehydrogenase (subunit B, SDHB), expressed significantly lower levels of PD-L1 mRNA when compared to sporadic cases (p = 0.0002) (**Figure 1C**). Visualization of the expression of *PD-L1* using data from the TCGA cohort selected on the presence of matched mutations (n = 72) confirmed a significant increase in *PD-L1* expression in the kinase signaling cluster compared to NAM (n = 3) (p = 0.0013), but not in the pseudohypoxia cluster (**Figure 1D**). In agreement with our cohort, the TCGA pseudohypoxia cluster shows decreased expression of *PD-L1* if compared to the kinase signaling cluster (p < 0.0001) (**Figure 1D**). We did not find differences in the expression of *PD-L2* in either of our cohorts (**Figure 1E-G**) or the mutation-matched TCGA tumor samples (**Figure 1H**).

**Figure 2:**
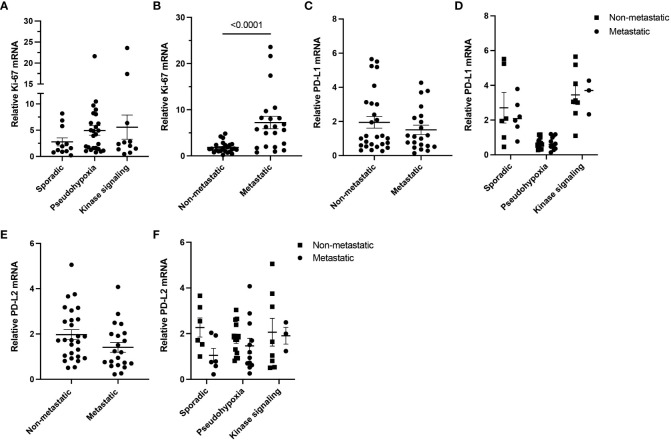
*Ki-67*, *PD-L1*, and *PD-L2* expressions in metastatic and non-metastatic PPGLs. **(A)** Expression of *Ki-67* measured in PPGL subtype clusters. **(B)** Expression of *Ki-67* in metastatic and non-metastatic PPGLs. **(C)** Expression of *PD-L1* in metastatic and non-metastatic PPGLs. **(D)** Expression of *PD-L1* in metastatic and non-metastatic tumors in PPGL clusters. **(E)** Expression of *PD-L2* in metastatic and non-metastatic PPGLs. **(F)** Expression of *PD-L2* in metastatic and non-metastatic tumors in PPGL clusters.

It has been suggested that *PD-L1* expression may correlate with metastatic behavior in PPGL, therefore, representing a marker for metastatic/locally advanced PPGL therapies. Based on this notion, we examined the expression of *PD-L1* and *PD-L2* in relation to metastatic status of tumor in our cohort. First, we evaluated level of Ki-67 mRNA, a marker of proliferation, and did not find any significant difference in the clusters of our cohort (**Figure 2A**). While *Ki-67* was significantly increased in metastatic samples (n = 21) compared to their non-metastatic counterparts (n = 27, p < 0.0001) (**Figure 2B**), neither *PD-L1* (**Figures 2C, D**) nor *PD-L2* (**Figures 2E, F**) were significantly altered in the context of the metastatic status. Overall, we did not observe any relation in expression of *PD-L1* and *PD-L2* to metastatic behavior of PPGL tumors.

## Discussion

Recent advances in tumor immunotherapy have highlighted their use as a potential tool to target metastatic/advanced tumors where ‘traditional’ therapies are ineffective and do not provide significant systematic and long-term antitumor effect. Despite breakthroughs in immunotherapy, such as the recognition of tumor immune escape mechanisms and characterization of immune checkpoints, still few patients with advanced/metastatic cancer respond to immune checkpoint inhibitors. Thus, tools to predict responders to therapy and maneuver the tumor immune microenvironment are needed for more successful clinical trials outcomes, including the focus on rare and metastatic cancers like PPGL.

PPGLs are recognized to have the highest rate of heritability within all tumor types, especially endocrine ones (17), and except surgery, there is currently no cure for patients with metastatic disease (18). The unique molecular landscapes of these tumors, however, can help predict the risk of metastatic behavior (19). Furthermore, understanding the tumor immune microenvironment in relation to oncogenic driver mutations could enhance our understanding of which patients are well suited for immunotherapy, while reducing the number of patients exposed to often ineffective, toxic, and costly treatments. Since potential immunotherapy biomarkers serves as one of several promising approaches to predict the response in patients (13, 14, 20–24), we focused on establishing the connection of PD-L1/PD-L2 to predisposing gene mutations and metastatic behavior in our representative cohort of PPGLs.

In our cohort, the pseudohypoxia cluster showed lower levels of *PD-L1* when compared to the sporadic and the kinase signaling clusters. Since *PD-L1* expression can represent a T cell-mediated inflamed tumor microenvironment (25–28), lower expression in the pseudohypoxia cluster most likely correlates to a lower T cell involvement/immune activation. A recent study on the tumor immune microenvironment of PPGLs is consistent with this notion since the lowest proportion of cytotoxic T-lymphocytes in PPGLs has been associated with *SDHB* mutations (29). This further suggests that reduced immune cells presence in tumors corresponds to higher risk of their metastatic behavior (29, 30). In our cohort, one patient with *SDHB* germline variant received immunotherapy (nivolumab and ipilimumab), besides other treatments, however, the patient had metastatic, progressive disease at the time of these therapies and very soon afterwards progressed, without indication of significant stabilization. Interestingly, we found similar results for *PD-L1* expression in non-*SDHB* mutated pseudohypoxia PPGLs, suggesting a broader application to the pseudohypoxia cluster. Contrary to these findings, Pinato et al. did not find a correlation between PPGL well known germline mutations in their case series and in the TGCA data set, concluding that pseudohypoxic signals may not be the sole drivers of PD-Ls expression. This cohort included 28% patients with germline mutations (cohort of 100 patients, 14% of cohort pseudohypoxia cluster, 14% of cohort kinase signaling cluster) (15). Given that only 18% of the cohort had positive PD-L1 staining, the lack of correlation between germline mutation and PD-L1 expression could be caused by the relatively small cohort or specific patient’s clinical characteristics. Here, we focused on tumors that already show clinical correlation in predicting metastatic behavior. In TCGA, we observed significant differences in *PD-L1* expression based on driver mutations, where only the kinase signaling cluster differed from NAM, which was reaffirmed by results from the cohort of our patients. Based on the expression of *PD-L1* in our patient cluster, sporadic cases and the kinase signaling cluster of PPGLs seem to be better suited for targeted anti-PD-1/PD-L1 therapies.

In our pseudohypoxia cluster where *PD-L1* expression was the lowest of all clusters, both metastatic (n = 13) and non-metastatic samples (n = 12) were included, pointing to the lack of expression effect correlating to the metastatic potential. Furthermore, we did not observe correlations in expression of *PD-L1* and *PD-L2* with malignancy status in patients with metastatic or non-metastatic tumors (n = 21 vs 27, respectively). However, we found a significant increase in *Ki-67* expression in our metastatic samples, which was previously described by Guo et al. (16), but we did not see correlation with *PD-L1* expression. We also did not find any correlation of *PD-L1* expression to the tumor type in our pseudohypoxia cluster where we were able to compare 14 PGL and 11 PCC. While other studies focused primarily on immunohistochemistry staining, for which conflicting results were reported using different PD-L1 antibodies (31–33), we measured mRNA expression. Yet Guo et al. found strong correlation of PD-L1 mRNA and protein expression with solid κ coefficient of 0.828 and high consistency (Pearson coefficient 0.753) (16). Thus, this cannot explain discrepancies between cohorts, and additional studies should be performed to provide more data on PD-L1 levels in patients with various PPGLs. We acknowledge that our cohort is too small to be fully representative of PPGL patients, and we did not include rarer gene mutations and additional clusters, as reported in other studies (11, 34, 35). However, despite rarity of these tumors, we have included a reasonable number of patients with metastatic disease to identify a possible connection between metastatic behavior and *PD-L1*/*PD-L2* expression. Nevertheless, more studies in these rare tumors and multi-institutional collaborative efforts could help increase the number of patients and provide a more robust and clinically relevant study.

In conclusion, our results demonstrate that the *PD-L1* expression is markedly different in PPGLs compared to normal adrenal medulla, and the expression may be linked to the genetic background of the tumor, suggesting that targeting the PD-1/PD-L1 pathway in certain PPGL clusters may lead to better outcomes, regardless of the metastatic status of the patient.

## Data availability statement

The original contributions presented in the study are included in the article/**Supplementary Material**. Further inquiries can be directed to the corresponding author.

## Ethics statement

The studies involving human participants were reviewed and approved by National Institutes of Health Institutional Review Board (NIH Protocol 00-CH-0093). Written informed consent to participate in this study was provided by the participants or participants’ legal guardian/next of kin.

## Author contributions

KHV, OU, and KP designed the study. KHV and OU performed the experiments. SG analyzed and processed the TCGA data. LM, ST, MP collected the patient data and samples. KHV, OU, SG, verified, analyzed, and interpreted the data. JN and KP supervised and administrated the project. All authors contributed to the article and approved the submitted version.

## Funding

This research was supported by the Intramural Research Program of the National Institutes of Health, *Eunice Kennedy Shriver* National Institute of Child Health and Human Development.

## Conflict of interest

The authors declare that the research was conducted in the absence of any commercial or financial relationships that could be construed as a potential conflict of interest.

The handling editor LL declared a past co-authorship with the author KP.

## Publisher’s note

All claims expressed in this article are solely those of the authors and do not necessarily represent those of their affiliated organizations, or those of the publisher, the editors and the reviewers. Any product that may be evaluated in this article, or claim that may be made by its manufacturer, is not guaranteed or endorsed by the publisher.
