# Supplementary material for: PD-L1 expression and association with genetic background in pheochromocytoma and paraganglioma
Source: Front Oncol. 2022 Nov 11;12:1045517. doi: 10.3389/fonc.2022.1045517 (PMC9691952; doi:10.3389/fonc.2022.1045517)
Supplement: Supplementary file 1 [file DataSheet_1.pdf]

Supplementary Material

1 Supplementary Tables

Supplementary Table 1: Characteristic of patient cohort

Abbreviations: PCC: pheochromocytoma; PGL: paraganglioma; P: primary; ML: metastatic lesion; Met: metastatic; Non-Met: non-metastatic; LLQ: lower left quadrant; F: female; M: male; N: noradrenergic; A: adrenergic; ND: not determined; MUL: multiplicity, REC: recurrent, #MIBG: Iobenguane I-131 treatment to lesion prior to surgery; ##C, R, I: chemotherapy, radiation therapy, immunotherapy treatment to lesion prior to surgery; * deceased at the time of publication

Patient ID	Primary tumor	Location	Tumor type	Variant status	Metastatic status	Sex	Age at time of surgery	Biochemical phenotype	Notes
1	PCC	Right adrenal	P	Sporadic	Non-Met	F	56.1	N	
2	PCC	Left adrenal	P	Sporadic	Non-Met	M	51.9	N	
3	PCC	Left adrenal	P	Sporadic	Non-Met	M	46.5	N	
4	PCC	Right adrenal	P	Sporadic	Non-Met	F	59.3	N	
5	PCC	Right adrenal	P	Sporadic	Non-Met	F	50.5	N	
6	PCC	Right adrenal	P	Sporadic	Non-Met	M	36	N	
7	PCC	Right adrenal	P	Sporadic	Met	M	61.7	ND	
8	PCC	Retroperitoneum	ML	Sporadic	Met	F	58.5	N	
*9	PCC	Peri-aortic	ML	Sporadic	Met	M	59	N	
10	PCC	Liver	ML	Sporadic	Met	F	47.4	N	
*11	PCC	Right adrenal	P	Sporadic	Met	M	67	N	
12	PCC	Liver	ML	Sporadic	Met	M	44	ND	
13	PCC	Carotid body	P	SDHB	Non-Met	F	57.6	ND	MUL
14	PGL	Para-aortic	P	SDHB	Non-Met	M	41.4	ND	
15	PGL	Carotid body	P	SDHB	Non-Met	F	15.4	ND	
16	PGL	Retroperitoneum	P	SDHB	Non-Met	F	15.8	N	REC
17	PGL	Retroperitoneum	P	SDHB	Non-Met	F	30.8	N	
18	PGL	Carotid body	P	SDHB	Non-Met	F	50	ND	
*19	PCC	Liver	ML	SDHB	Met	F	34.8	N	#MIBG
*20	PGL	Liver	ML	SDHB	Met	M	48.3	N	
*21	PGL+PCC	Left adrenal	P	SDHB	Met	M	40.5	ND	MUL
22	PGL	Paracaval	P	SDHB	Met	M	15.1	N	MUL
23	PGL	Retroperitoneum	P	SDHB	Met	M	16.1	ND	MUL
*24	PGL	Extracranial	ML	SDHB	Met	M	35.3	ND	##C, R, I
25	PGL	Epidural T3-T4 lesion	ML	SDHB	Met	M	47	N	
26	PGL	Carotid body	P	SDHB	Met	M	46	N	MUL
27	PCC	Left adrenal	P	VHL	Non-Met	M	5.5	N	
28	PGL	Retroperitoneum	P	VHL	Non-Met	M	7.3	N	

29	PGL	Paracaval	P	VHL	Non-Met	F	13.5	N	
30	PCC	Left adrenal	P	VHL	Non-Met	M	11.4	N	
31	PCC	Right adrenal	P	VHL	Non-Met	M	18.7	N	MUL
32	PCC	Left adrenal	P	VHL	Non-Met	M	12.1	N	MUL
33	PCC	Left adrenal	P	VHL	Non-Met	M	14.1	ND	MUL
34	PCC	Right adrenal	P	VHL	Met	F	14	N	MUL
35	PGL	Left adrenal	P	EPAS1	Met	F	11.2	N	MUL, REC
36	PCC	Right adrenal	P	EPAS1	Met	M	17.3	N	MUL
37	PGL+PCC	Left adrenal	ML	EGLN1	Met	F	48	N	
38	PCC	Right adrenal	P	RET	Non-Met	F	26.7	N	
39	PCC	Right adrenal	P	RET	Non-Met	M	29.1	N	
40	PCC	Left adrenal	P	RET	Non-Met	F	24	A	
41	PCC	Adrenal, bilateral	P	RET	Non-Met	M	43.7	N	MUL
42	PCC	Abdominal LLQ	ML	RET	Met	F	30	N	REC
43	PCC	Left adrenal	P	RET	Met	F	44	N	
44	PCC	Adrenal, bilateral	P	RET	Met	F	22	N	
*45	PCC	Right adrenal	P	NF1	Non-Met	M	17	N	
46	PCC	Right adrenal	P	NF1	Non-Met	M	26.6	N	
47	PCC	Right adrenal	P	NF1	Non-Met	M	64.8	N	
48	PCC	Adrenal, bilateral	P	NF1	Non-Met	F	63.7	N	REC
